# Design and Fabrication of an Integrated Hollow Concave Cilium MEMS Cardiac Sound Sensor

**DOI:** 10.3390/mi13122174

**Published:** 2022-12-08

**Authors:** Bo Wang, Pengcheng Shi, Yuhua Yang, Jiangong Cui, Guojun Zhang, Renxin Wang, Wendong Zhang, Changde He, Yirui Li, Shuotong Wang

**Affiliations:** State Key Laboratory of Dynamic Measurement Technology, School of Instrument and Electronics, North University of China, Taiyuan 030051, China

**Keywords:** heart-sound sensor, MEMS, cilium, broadband, high sensitivity

## Abstract

In light of a need for low-frequency, high sensitivity and broadband cardiac murmur signal detection, the present work puts forward an integrated MEMS-based heart sound sensor with a hollow concave ciliary micro-structure. The advantages of a hollow MEMS structure, in contrast to planar ciliated micro-structures, are that it reduces the ciliated mass and enhances the operating bandwidth. Meanwhile, the area of acoustic-wave reception is enlarged by the concave architecture, thereby enhancing the sensitivity at low frequencies. By rationally designing the acoustic encapsulation, the loss of heart acoustic distortion and weak cardiac murmurs is reduced. As demonstrated by experimentation, the proposed hollow MEMS structure cardiac sound sensor has a sensitivity of up to −206.9 dB at 200 Hz, showing 6.5 dB and 170 Hz increases in the sensitivity and operating bandwidth, respectively, in contrast to the planar ciliated MEMS sensor. The SNR of the sensor is 26.471 dB, showing good detectability for cardiac sounds.

## 1. Introduction

Auscultation is one of the routine diagnostic techniques for clinical cardiovascular diseases. Before the 19th century, doctors rested their ears against a patient’s chest for “direct hearing”. It was not until 1816 that Laennec, a French physician, developed a wooden stethoscope, thus enabling “indirect auscultation” and forming disciplines including cardiac auscultation [1]. Cardiac sound signals respond to the mechanical movement and comprehensive status of the cardiac and cardiovasculature systems, including the pathological and physiological traits of various cardiac parts, as well as relevant interactions [2]. At present, there is still a problem of delayed detection in the diagnosis of cardiovascular disease by ECG. For example, in the detection of coronary cardiac disease, a change in the ECG signal can only be caused when the coronary artery occlusion rate is above 70%. In actual clinical tests, the cardiac sound signal can be changed when the obstruction reaches 25% [3]. Since the cardiac sound signal has a wide-distribution frequency range (distributed in 20~800 Hz), there are low-frequency components (below 100 Hz), mid-frequency components (100~200 Hz), and high-frequency components (above 200 Hz) [4]. Therefore, higher requirements are proposed for the working bandwidth and sensitivity of electronic stethoscopes. The research and development of high-performance cardiac sound sensors have become the core of electronic stethoscope development [5].

After constant progress and enhancement regarding medical auscultation, 3M Littmann in the USA made a prominent breakthrough in 2000, namely the first attempt to develop an electronic stethoscope for auscultation. Alongside the ongoing scientific development and improvement, the stethoscope is becoming intelligent, electronic, and mobile. In addition, Bifulco et al. proposed a PVDF sensor that allows simultaneous recording of respiration, Seismocardiogram (SCG), and heart sounds in 2014 [6]. Liu et al. described an epidermal mechano-acoustic sensor based on soft electronics in 2016 [7]. Jain et al. focused on extracting heart sounds from SCG signals, thus demonstrating the possibility of using accelerometers as heart-sound sensors in 2016 [8].Anumukonda, M. et al. put forward a high-density microphone-based heart-sound sensor in 2016 [9]. Afattah et al. invented a cardiac acoustic sensor utilizing a piezoelectric PVDF thin film in 2017 [10]. Moreover, Choudhary et al. devised a method for extracting basic heart sounds (HSs) from SCG signals to achieve a good location of HS waves S1 and S2 in one SCG cycle without the use of a reference ECG cycle in 2018 [11]. Martinek et al. fabricated an advanced interferometric heart-sound sensor in 2018 with the application of optical fiber [12]. Lee et al. proposed a novel skin-compliant device based on triaxial accelerometers with 800 Hz bandwidth, which proved capable of recording various physiological activities, such as respiration, cardiac activity (including heart sounds), swallowing, and vocal-fold vibrations in 2019 [13]. Ha et al. presented an E-tattoo sensor capable of recording ECG, SCG, and heart sounds in 2019 [14]. Gupta et al. developed a monolithic integrated sensor that featured a triaxial accelerometer for monitoring body motion, respiration, and SCG, and a piezoelectric sensor for simultaneous recording of heart and lung sounds in 2020 [15]. A piezoelectric lead zirconate titanate sensor designed by Andreozzi et al. was proposed for use in cardiographic techniques in 2021. It allows simultaneous recording of breathing, infrasound heart vibrations, and heart sounds from a single location in the chest [16]. Centracchio et al. developed a new technique for measuring the local force on the chest wall generated by the mechanical activity of the heart through cardiography in 2022 [17]. Andreozzi et al. recorded heart-induced vibrations in the chest wall through cardiography using a special force sensor, which was shown to be able to simultaneously monitor breathing, heart sounds, and infrasonic heart vibrations from a single point of contact on the chest in 2022 [18]. However, the common piezoelectric thin-film electronic stethoscope cannot obtain much information about the cardiac sound signal because of their poor low-frequency response and the primary distribution of the main components of the cardiac sound signal in the low-frequency range, which makes the investigation of the recognition and feature extraction of heart acoustic signals extremely challenging.

With the advancement and progress of MEMS technology, the use and promotion of precision biomimicry of biological microstructures has become technically feasible in the medical field. Using MEMS technology, Zhang Guojun et al. developed a bionic sensing unit for identifying cardiac sounds in 2019, in which the piezoresistors and beams were used as the core, and the piezoresistive effect was used to establish a signal-conversion bridge between mechanical energy and electrical energy. This was a beneficial attempt to enhance the sensitivity and SNR of the cardiac sound sensor, implying diversified progress in the techniques for cardiac acoustic auscultation [19]. MEMS sensors have been shown to accurately measure heart-sound signals. In this study, we investigated a new type of MEMS sensor that is also capable of capturing heart sounds, thus enriching the information obtained through MEMS technology. In particular, the new MEMS sensor integrated with hollow concave cilia proved to be a suitable device for collecting heart-sound signals, providing a new sensor design method. The fixed ear on the sensor can be fixed to the human body, reducing friction interference generated by handheld sensors, while the acoustic impedance pack design allows better perception and acquisition of heart-sound signals [20]. The 3M Littmann electronic stethoscope is a relatively mature electronic stethoscope on the market. The ability to collect heart-sound signals was compared between our MEMS sensor and a 3M electronic stethoscope to prove the reliability of sensing and acquiring heart-sound signals.

## 2. Materials and Methods

### 2.1. Principle and Structure

**Figure 1:**
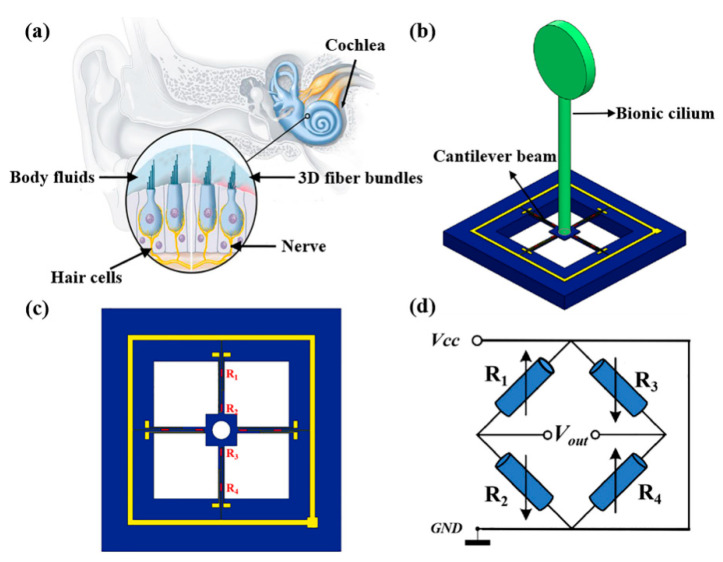
(**a**) Mechanism of human hearing. (**b**) Micro-structure of biomimetic MEMS. (**c**) Schematic diagram of cantilever beam structure. (**d**) Wheatstone bridge.

As depicted in Figure 1a, the MEMS-based cardiac sound sensor has a cilium–beam architecture that primarily biomimics the structural pickup mechanism of 3D hair-cell fiber bundles in the human aural system. The cardiac sound signal is transmitted to the surface of the thoracic cavity through human tissue and is perceived by the probe of the proposed sound sensor. Acting on the rigid bionic cilium of the sensor causes the rigid bionic cilium to swing, and the swing of the cilium drives the connected cantilever beam to twist. The novel biomimetic MEMS micro-structure composed of cilium fabricated using 3D printing technology and a high-precision cantilever fabricated using the MEMS process are shown in Figure 1b. Combining the piezoresistive effect and MEMS process technology, a variable resistor is implanted in the area where the linear stress of the cantilever changes the most as illustrated in Figure 1c, and the Wheatstone bridge is formed, as presented in Figure 1d (*V_CC_* indicates the input voltage for the bridge and *V_out_* indicates the output voltage).

**Figure 2:**
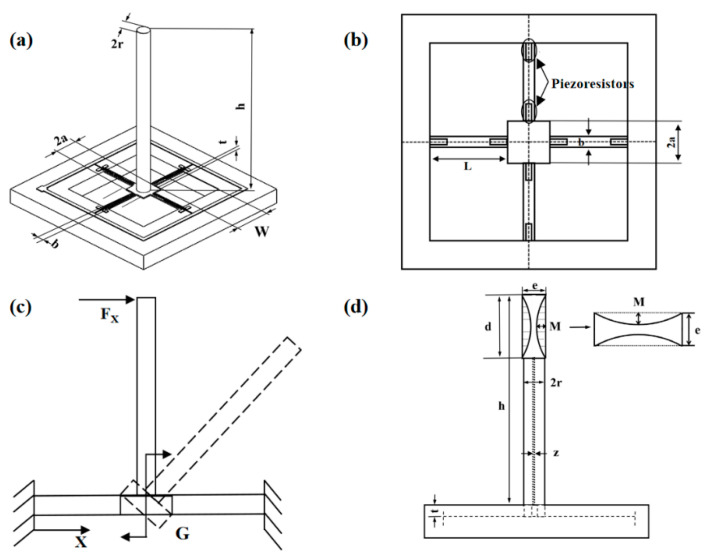
(**a**) Micro-structure model for the sensor. (**b**) Model for piezoresistor on beams. (**c**) Mechanical analysis of the micro-structural model. (**d**) Micro-structure model of the hollow-concave cilium.

When the sound wave applies to the cilium, the cilium swings under force, and the beams connected with the cilium deform. Resistance values of the variable resistor on the beams change because of the force as shown in Figure 2c. *R*_1_, *R*_2_, *R*_3_ and *R*_4_ form the Wheatstone bridge [21,22]. Finally, via the Wheatstone bridge, the transformation of mechanical distortion into voltage output is possible, and the acousto-electric conversion of the cardiac sound signal is realized. Where the cilium is not imposed with any acoustic wave, the Wheatstone bridge becomes a balanced circuit. The computational formula for the bridge’s output voltage in an equilibrium state is shown in Formula (1):

(1) $$V_{out}=\frac{\left(R_{1}R_{3}\right)-\left(R_{2}R_{4}\right)}{\left(R_{1}+R_{2}\right)\left(R_{3}+R_{4}\right)}V_{cc}$$

When the sound wave applies to the cilium, the cantilever beam is deformed. The computational formula for the bridge’s output voltage in the current case is presented in Formula (2):

(2) $$V_{out}=\frac{\left(R_{1}+\Delta R_{1}\right)\left(R_{3}+\Delta R_{3}\right)-\left(R_{2}-\Delta R_{2}\right)\left(R_{4}-\Delta R_{4}\right)}{\left(R_{1}+R_{2}+\Delta R_{1}-\Delta R_{2}\right)\left(R_{3}+R_{4}+\Delta R_{3}-\Delta R_{4}\right)}V_{cc}$$

The respective voltage resistor values in this Formula satisfy *R*_1_ = *R*_2_ = *R*_3_ = *R*_4_ = *R*, and the corresponding variation amount for resistance values is Δ*R*_1_ = Δ*R*_2_ = Δ*R*_3_ = Δ*R*_4_ = *R* and Δ*R* ≪ *R*. Hence Formula (1) can be reduced to Formula (3).

(3) $$V_{out}=\frac{\Delta R}{R}V_{CC}$$

The resistivity-alteration-induced relative variation of resistance can be indicated by:

(4) $$\frac{\Delta R}{R}=\pi_{l}\sigma_{l}$$

In Formula (4), $\sigma_{l}$ means the longitudinal stress on each piezoresistor, $\pi_{l}$ refers to the longitudinal piezoresistive coefficient of each piezoresistor. With Formulas (3) and (4), the output voltage is proportionally correlated with the variation in piezoresistors’ resistance. Owing to cantilever beam stress, greater stress changes imply greater piezoresistance changes as well as higher cardiac sound sensor sensitivity.

The mechanical analysis of the micro-structural model is shown in Figure 2c, where F_X_ denotes the force applied to the cilium in the X direction. In line with theoretical mechanics and material mechanics principles, the force analysis of each cantilever beam is available [23]:

(5) $$\sigma_{\left(x\right)}=\pm \frac{L^{2}+3aL-3x\left(a+L\right)}{\frac{2}{3}Wt^{2}\left(L^{2}+3aL+3a^{2}\right)}PSh_{0}\pm \frac{PS}{Wt}$$

In

(6) $$S=\pi \left(1+M^{2}\right)+\pi r\left(h-d\right)$$

For

(7) $$h_{0}=\frac{d}{2}+\frac{\left(h-d\right)}{2}$$

In Formula (5), *P* and *S* refer separately to the external acoustic pressure of and force area on the acoustic signal receiving cilium, and *h*_0_ stands for the cilium structure barycenter. The expressions of surface *S* and center of gravity *h*_0_ of the concave cilia are shown in Formulas (6) and (7). *L* is the length of the micro-structure cantilever beam, *t* is the thickness of the micro-structure cantilever beam, *W* is the width of the micro-structure cantilever beam, and 2*a* is the length of the middle block side of the micro-structure cantilever beam. Although directly proportional to the area for acoustic signal acquisition, the stress on the cross beam shows the inverse proportion to the cantilever beam width and thickness. The bionic cilium is a vital structure for the reception of cardiac sound signals by the MEMS-based sensor. According to Formula (5), the stress on the cantilever beam shows a positive correlation with the stress area of cilium [23].

**Figure 3:**
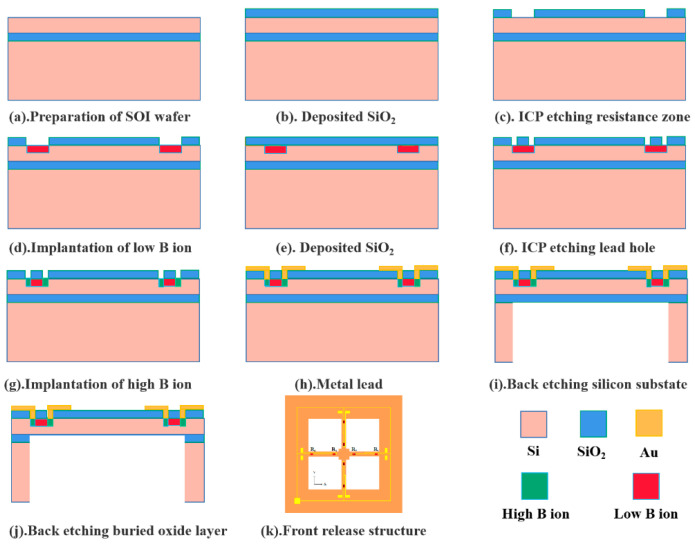
Schematic diagram of the main process flow of the sensor micro-structure.

Thus, the MEMS cardiac sound sensor’s sensitivity is promoted by enlarging the cilium’s force area. Through the incorporation of particular actual conditions into the process flow design, a design strategy is formulated, which consists of processes such as lithography, doping, bonding, oxidation, dry/wet etching, implantation of ions, metal sputtering, deposition, scribing, and cleaning [24]. Figure 3 illustrates the overall process flow. Table 1 presents the structural parameters in the micro-structure.

The natural frequency of sensor micro-structure shows an association with micro-structure size as seen in Figure 2d. For the reception and detection of cardiac sound signal scope, the sensor’s working frequency band is a direct impact factor, which becomes wider as the natural frequency of the micro-structure heightens. Since the cardiac sound signal has a wide-distribution frequency range (distributed in 20~800 Hz), there are low-frequency components (below 100 Hz), mid-frequency components (100~200 Hz), and high-frequency components (above 200 Hz) [25]. Hence, for the valid reception of most pathologic cardiac murmurs, the heart-sound sensor must have a high operating frequency band. The computational formula for the micro-structure’s natural frequency f is shown in Formula (8):

(8) $$f=\frac{1}{2\pi }\sqrt{\frac{k}{m}}$$

where *k* represents the beam rigidity and m represents the cilium mass. According to Formula (8), the working frequency band of the micro-structure will decrease as cilium mass increases. Hence, for improving the sensitivity and working frequency band of the micro-structure, it is necessary to optimize it. Thus we improve the working frequency band of the micro-structure by reducing the mass of cilium and improve the sensitivity of the micro-structure by increasing the force area of cilium receiving sound waves. In addition, the natural frequency of the cilium structure is associated with Young’s modulus in the material the cilium is made of.

(9) $$f=\frac{\pi a}{16L^{2}}\sqrt{\frac{E}{\rho }}$$

In Formula (9), ρ means the material density in the cilium structure and *E* indicates Young’s modulus in the cilium structure. As Young’s modulus increases, the natural frequency of the micro-structure increases. Therefore, for improving the natural frequency and performance of the cardiac sound sensor, cilium material must be carefully chosen based on two requirements: (1) the cilium material density must be close to human tissue density; (2) Young’s modulus in the cilium material must be relatively large. In our research, photosensitive resin was selected as the cilium material, and Table 2 demonstrates its precise material properties.

**Figure 4:**
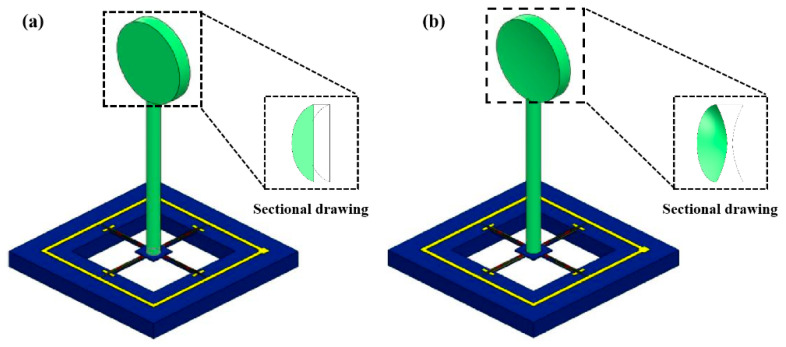
(**a**) Integrated micro-structure model of the planar cilium. (**b**) Integrated micro-structure model of the hollow concave cilium.

Under the above theory, to realize the wide-band and low-frequency high-sensitivity features of the cardiac sound sensor, this study designed a hollow concave cilium. At an identical size, in contrast to the MEMS cardiac sound sensor with a planar bionic cilium (Figure 4a), the hollow concave cilium micro-structure (Figure 4b) allows the cardiac sound sensor to have a broader frequency band and high sensitivity to low frequencies. While the heart-sound sensor collected heart sounds, the subject lay supine on the platform and breathed naturally. The signal acquisition location of the heart-sound sensor is the mitral valve auscultation area.

### 2.2. Encapsulation Method

**Figure 5:**
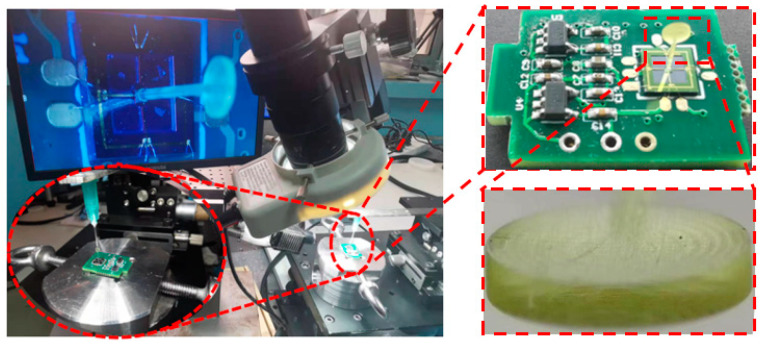
Secondary integration of sensor micro-structure.

An adhesive (UV-cured) is utilized to secondarily integrate the micro-structures of bionic cilium and sensor. The bionic cilium is made by 3D-printing technology, and the main material is resin. The 3D-printed-resin biomimetic cilium was integrated with the central mass of the cantilever micro-structure using UV-cured adhesive through microscope platform observation. The preparation of the micro-structure of the sensor is shown in Figure 5.

**Figure 6:**
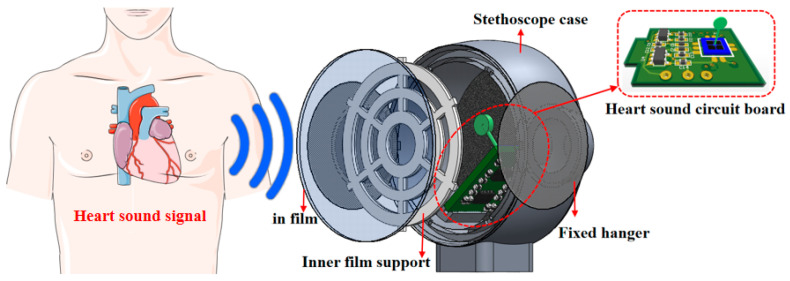
MEMS cardiac sound sensor acoustic encapsulate matching diagram.

The MEMS cardiac sound sensor acoustic encapsulate matching diagram is shown in Figure 6. The acoustic propagation theory in the 3-layer media holds that there is an enlargement of the reflection coefficient when the sound is input on the boundary between two differing media. Acoustic energy transmission, on the other hand, shows reduction as the impedance difference becomes greater. The reflection between the stethoscope and the skin intensifies as the two are in contact, leading to less sound energy transmission, as well as stronger attenuation [26]. Therefore, in order to maximally perceive and acquire the cardiac sound signal, the cardiac sound transmitted through the human tissue can be transmitted to the MEMS-based sensor for heart sounds without significant attenuation. Additionally, the concave structure of the cilium is parallel to the waterproof and sound-transmitting film (the main material is e-PTFE) to ensure the maximum cilium area for acoustic signal acquisition. Moreover, the medical silicone oil (20 cst) packaging material is selected to match the soft-tissue acoustic impedance traits in humans, so as to match the acoustic impedance of the sensor in the test environment. The velocities of sound and the characteristic acoustic impedances for various media can be found in Table 3.

(10) $$T=\frac{4Z_{1}Z_{3}}{\left(Z_{3}+Z_{1}{)}^{2}{cos}^{2}(k_{2}Y\right)+\left(Z_{2}+Z_{1}Z_{3}/Z_{2}{)}^{2}{sin}^{2}(k_{2}Y\right)}$$

where, *Z*_1_, *Z*_2_, and *Z*_3_ separately represent the acoustic eigen impedances for a 3-layer medium comprising the human soft tissue, the porous e-PTFE (expanded polytetrafluoroethylene) membrane, and the medical silicone oil. *k*_2_ denotes the quantity of acoustic waves transmitted via the e-PTFE medium, and Y denotes the thickness of the e-PTFE medium. As is clear from Formula (10), the acoustic transmission coefficient is capable of approximating 1 only in case the acoustic eigen impedances in the 3-layer medium are mutually approached, or in case the intermediate e-PTFE medium is sufficiently thin [27].

**Figure 7:**
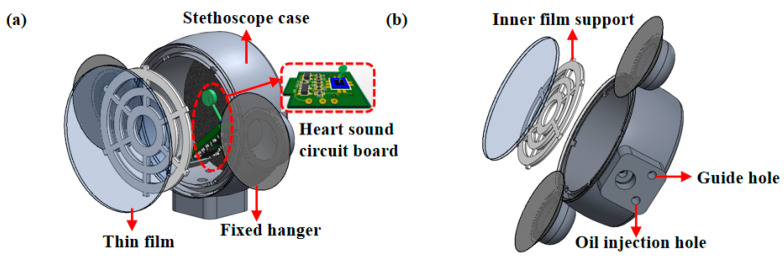
(**a**) Design of MEMS cardiac sound sensor probe. (**b**) MEMS cardiac sound sensor probe design side view.

Probe packaging architecture is designed for the MEMS-based heart sound sensor, with an emphasis on its pickup traits and the method of acoustic matching packaging. Additionally, the sensor probe is mainly composed of a stethoscope shell, waterproof and sound-permeable film, inner support of the film, fixed ear, oil injection hole, and air guide hole. On the premise of sealing the waterproof sound-permeable film, the film internal support structure was added and the film internal support was placed for auscultation, as illustrated in Figure 7a,b. In addition, the inner support of the film can also enhance the stiffness of the sound permeable film, so as to improve the natural frequency of the acoustically permeable film and evade its impact on the cardiac sound auscultation process. The medical silicone oil (20 cst) matches with the acoustic impedance of the sensor, aiming to lower the sound loss during the process of cardiac sound auscultation, improve the auscultation effect, and reduce the loss of cardiac sound distortion and weak cardiac murmur.

**Figure 8:**
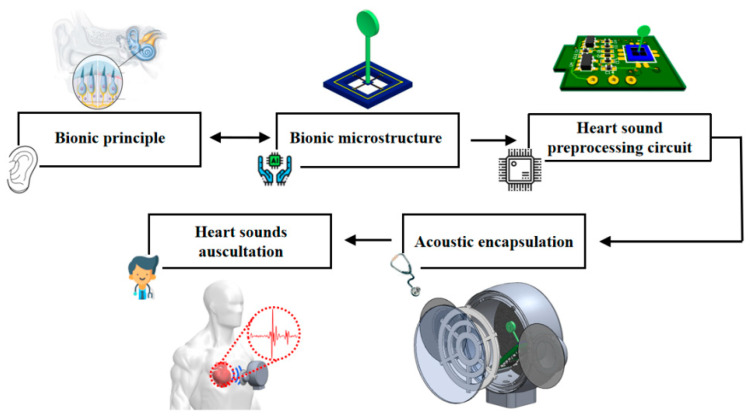
Overall system design block diagram.

**Figure 9:**
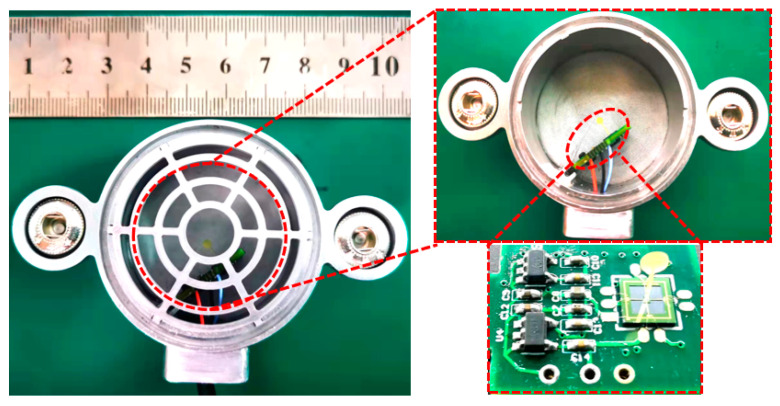
Overall packaging of MEMS cardiac sound sensor.

Fixed ears are sited on both sides of the stethoscope shell to fix the sensor probe at the cardiac sound auscultation position on the surface of human skin, so as to reduce the shaking noise caused by arm shaking when doctors hold the stethoscope probe and the friction noise resulting from friction between the sensor probe and the surface of human skin. Cardiac sound signals are transmitted to the surface of the thoracic cavity through human tissue and are sensed by the probe of the proposed heart sound sensor. The block diagram for overall system design is presented in Figure 8, and the encapsulation illustration of the sensor can be found in Figure 9.

## 3. Results

### 3.1. Simulation and Results

For determining the optimal cilium structural size and improving the performance of the cardiac sound sensor, COMSOL was used to parameterize the cilium size and architecture. In the model of integrated hollow concave cilium architecture displayed in Figure 2d, *r*, *h*, *d* and *e* refer, respectively, to the radius, height, diameter, and width of the hollow concave cilium; M represents the concave depth; and z stands for the hollow diameter. Due to the fabrication technical limitations, the scope of optimization for the cilium architecture size is 0.14 mm ≤ *r* ≤ 0.17 mm, 2.5 mm ≤ *h* ≤ 5.5 mm, 1.4 mm ≤ *d* ≤ 2.3 mm, 0.33 mm ≤ *e* ≤ 0.42 mm, and 0.01 mm ≤ *z* ≤ 0.08 mm.

**Figure 10:**
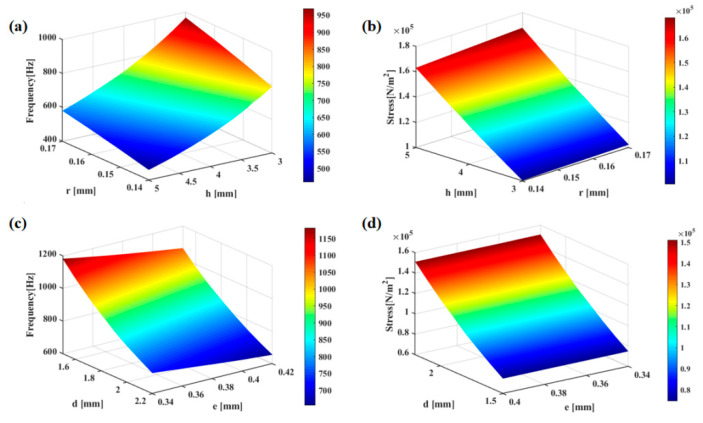
(**a**) Effects of radius r and height h on the natural frequency. (**b**) Effects of height r and radius h on the maximum beam stress. (**c**) Effects of the concave diameter d and the concave cilium width e on the natural frequency. (**d**) Effects of concave diameter d and concave cilium width e on the maximum stress on the beam.

**Figure 11:**
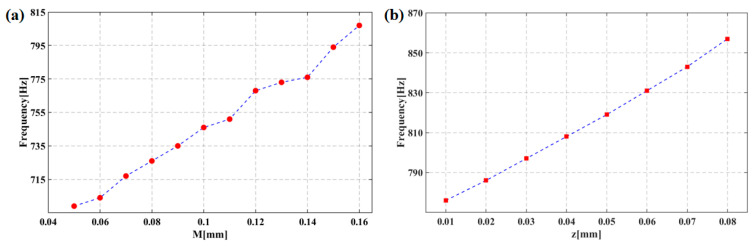
(**a**) Effects of the concave depth M on the natural frequency. (**b**) Effects of the hollow diameter z on the natural frequency.

An iterative technique was employed to conduct a simulation using FEA (finite element analysis). Figure 10a,c depict the enhancement of natural frequency with increasing *r* and its weakening with increasing *h*, *d* and *e*. According to Figure 10b,d, weakening of maximum stress is noted on the cantilever beam with increasing *r* and *h*, while as e rises, reduction of stress is observed. According to Figure 11a,b, with increasing *M* and *z*, the natural frequency also exhibits an increase.

With the consideration of practical demands on sensitivity and working frequency band, the dimensions of the concave hollow cilium micro-structure were determined as follows: cilium height was 4.9 mm; cilium radius was 0.165 mm; concave radius was 1 mm; overall concave width was 0.34 mm; concave depth was 0.12 mm, and hollow diameter was 0.05 mm.

**Figure 12:**
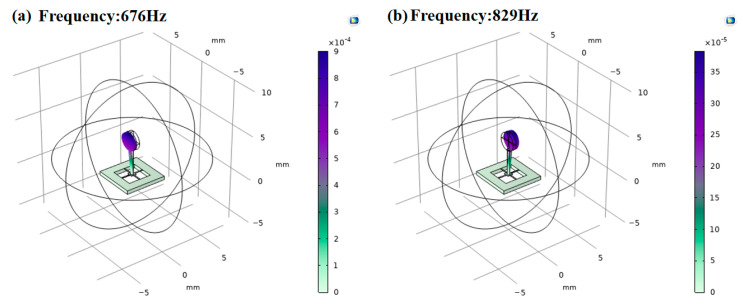
(**a**) Natural frequency in planar micro-structures. (**b**) Natural frequency in hollow concave micro-structures.

Based on the simulation, we determined the specific size of the hollow concave cilium. The micro-structure’s performance at this size needs to be simulated and analyzed for verifying design feasibility. Because the working environment of the cilium is medical silicone oil (20 cst), wet-mode simulation of the micro-structure was performed. With the same cilium size, the natural frequency for the planar ciliary micro-structure in medical silicone oil is as seen in Figure 12a, while that for the concave hollow ciliary micro-structure in medical silicone oil is as seen in Figure 12b. For the micro-structure, its natural frequency in a medical silicone oil environment was 829 Hz, satisfying the cardiac sound sensor’s requirements for operating frequency range. COMSOL simulation results showed that the optimized bionic micro-structure broadens the sensor’s working frequency band.

**Figure 13:**
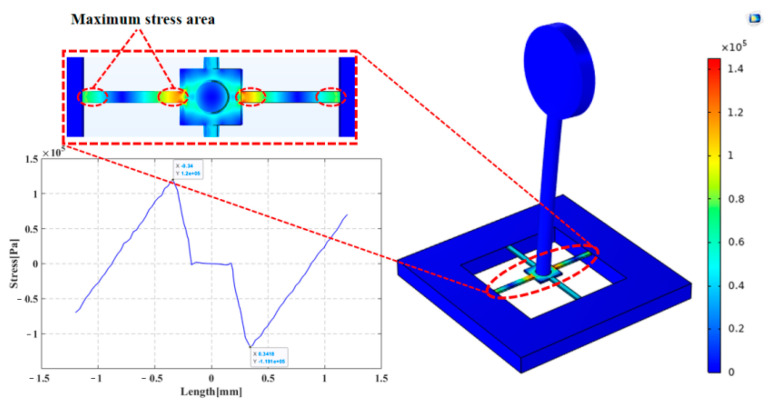
Centralized stress simulation of the cantilever beam micro-structure.

Furthermore, a stress simulation on the cantilever beam was performed. The X-directional stress magnitude variation was derived for the cantilever beam by applying 1 Pa acoustic pressure to the cilium in the X-direction [23]. Figure 13 pinpoints the maximum linear stress concentration area of the cantilever beam, where the maximal stress can be found from the cantilever beam’s junctions with the central mass block and with the support frame. To endow the sensor with low-frequency high sensitivity, the piezoresistors should be laid out at the location of maximal stress. For the hollow concave cilium micro-structure, its cantilever beam stress graph is displayed in Figure 13, where maximum stress of 1.1996 × 105 N/m^2^ is required for the cilium micro-structure’s integration. Because the simulation analysis shows that hollow cilium has a wide working frequency band and high sensitivity, the hollow cilium structure is designed feasibly.

### 3.2. Experiments and Results

**Figure 14:**
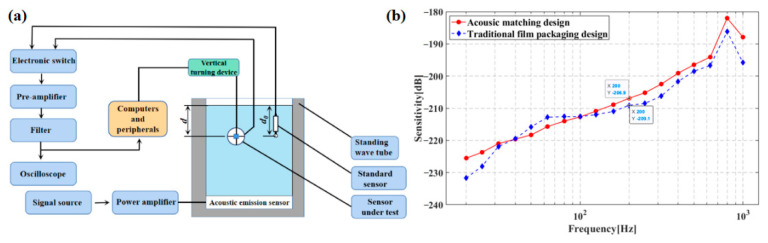
(**a**) Principle of calibrating standing-wave tube. (**b**) Acoustic encapsulate sensor low-frequency linear curve.

The principle of the system for calibrating standing-wave tubes is seen in Figure 14a. The system consists of the voltage source, signal generator, power amplifier, standing wave barrel, transmitting transducer, standard sound sensor, and oscilloscope. Sound is generated by a signal generator, and the signal is amplified by the power amplifier and transmitted to the standing-wave barrel, and then converted into a standing wave by the transmitting transducer. The sound signal received by the MEMS heart-sound sensor can be read out by the oscilloscope. The sensitivity of the bionic MEMS heart-sound sensor was tested in a standing-wave bucket by comparison calibration of the voltage signal of the standard sound sensor and MEMS heart-sound sensor. The standing-wave bucket contains a standing wave-sound field. The frequency of calibration is 1/3 times octave, based on the acoustic pressure distribution principle for the stationary wave acoustic field. Moreover, the sensitivity *M_p_* of the measured sensor is expressed as Formula (11) [25]. Figure 14b is the low-frequency linear curve of the acoustic encapsulated sensor, which shows the influence of the inner support of the encapsulated waterproof sound-permeable membrane on the low-frequency linearity of the sensor.

**Figure 15:**
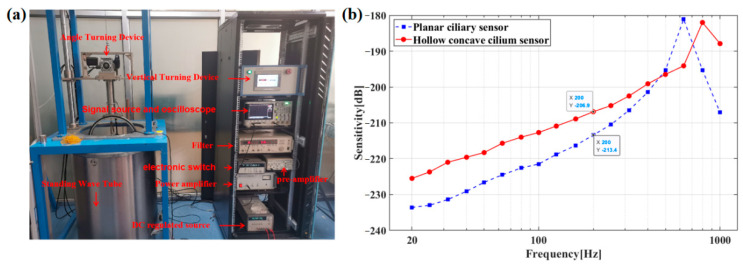
(**a**) Calibration system for the standing-wave tube. (**b**) Sensitivity curves of MEMS cardiac sound sensor with integrated planar cilium and with integrated hollow concave cilium.

Additionally, following the comparative calibration principle, the sensitivity assessment for MEMS-based cardiac sound sensors was accomplished with the standard sensor calibration system. The sensor under test and the standard sensor were put in water of an identical depth in the standing-wave tube so that the planar acoustic signals could be received from the acoustic emission sensor at the standing-wave tube’s bottom. The acoustic information sent out from the acoustic emission sensor could then be enlarged by a power amplifier and transferred to the acoustic emission sensor. Finally, the standard sensor received and converted acoustic information to a voltage signal and exhibited the information on an oscilloscope. Figure 15a shows the test system for the foregoing tube calibration scheme. The frequency of calibration is 1/3 times octave, in accordance with the acoustic pressure distribution principle for the stationary-wave acoustic field. The sensitivity *M_p_* of the measured sensor is expressed as Formula (11) [25]:

(11) $$M_{p}=M_{0}+20lg(\frac{e_{x}}{e_{0}}\frac{sinkd}{coskd})$$

where *M*_0_ means the sensitivity level in the standard sensor (*M*_0_ = −170 dB); *e_x_* and *e*_0_ indicate the open circuit voltages in the MEMS-based cardiac sound sensor and standard acoustic sensor, respectively; *d* represents the distance from the standing-wave tube’s water surface to the MEMS-based cardiac sound sensor; and k denotes the number of waves, where *k* = 2πf/c, with f representing the excitation signal frequency and c representing the sound velocity. The sensitivity curves of MEMS cardiac sound sensors with integrated planar and hollow concave cilia are displayed separately in Figure 15b.

### 3.3. Performance Results

**Figure 16:**
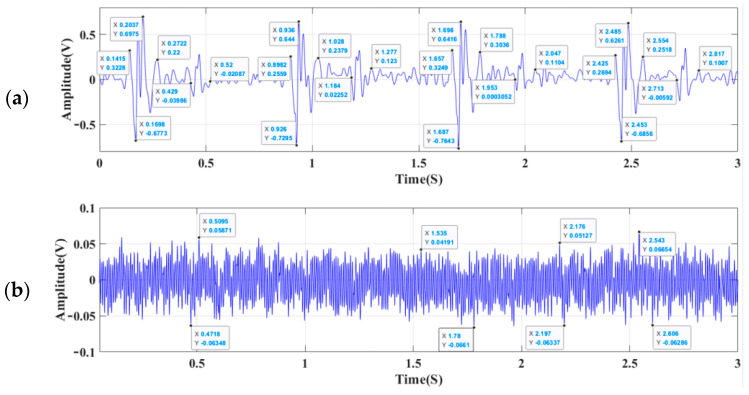
(**a**) The cardiac sound test results for a 3M Littmann 3200 stethoscope. (**b**) The static noise for a 3M Littmann 3200 electronic stethoscope.

**Figure 17:**
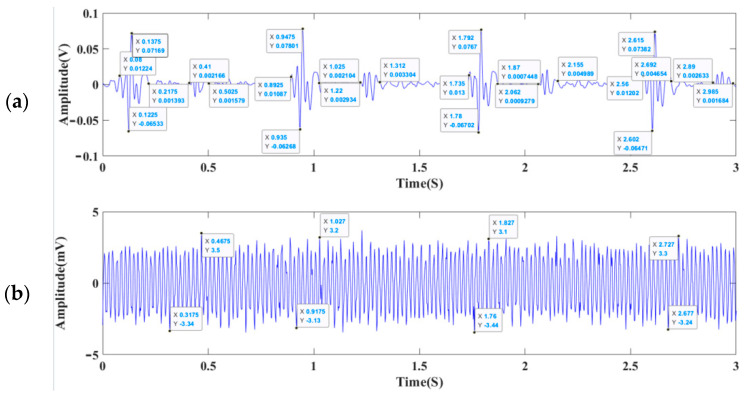
(**a**) The cardiac sound test results of the MEMS cardiac sound sensor. (**b**) The static noise of the MEMS cardiac sound sensor.

Figure 16a,b describe the heart-sound signal and static noise test results of the 3M electronic stethoscope respectively, while Figure 17a,b show the heart-sound signal and static noise test results of the sensor designed in this paper. As is clear, the SNR reaches 26.471 dB in the MEMS cardiac sound sensor and 21.233 dB in the 3M electronic stethoscope. With a 5.238 dB higher SNR than the control, the proposed MEMS cardiac sound sensor is proven feasible for collecting acoustic signals of the heart. As also corroborated by the experimental results, the present micro-structure design scheme is reasonable. The SNR can be calculated using Formula (12) [25], and Table 4 details the experimental acoustic signal data of the heart:

(12) $$SNR=20log\left(\frac{V_{s}}{V_{n}}\right)$$

where *Vs* denotes the peak cardiac sound output and *V_n_* refers to the stethoscope background noise.

## 4. Discussion

To fulfill the requirements of high sensitivity at low frequencies and wideband cardiac murmur signal detection at the same size, the present work put forward that a MEMS heart-sound sensor with an integrated hollow ciliary architecture is distinctly superior in several aspects. By comparing the two groups of experimental data, it can be seen that the index parameters of the MEMS electronic stethoscope and 3M electronic stethoscope are consistent in the time domain, and the time ratio between systolic and diastolic periods is about 3:5, which is consistent with medical theory and the detection performance of heart-sound signals of the same type stethoscope [28,29,30,31,32]. The comparison of the two methods of heart-sound signal acquisition further indicates the reliability of the MEMS electronic stethoscope designed in this paper. Compared with flat cilia, concave cilia have a good performance advantage, but because of its complexity and high precision requirements, it poses a challenge for the mass manufacturing of a MEMS electronic stethoscope of this type. The processing and design of biomimetic cilia provide a new direction for subsequent research.

## 5. Conclusions

To conclude, this study designed a MEMS cardiac sound sensor with a hollow concave cilium. First, in comparison with the integrated planar ciliated MEMS cardiac sound sensor, the concave hollow ciliary architecture has a sensitivity of up to −206.9 dB at 200 Hz, and its working bandwidth and sensitivity are increased by 170 Hz and 6.5 dB, respectively, realizing high sensor sensitivity at low frequencies, as well as broadband traits. Secondly, apart from lowering the cardiac sound distortion loss and faint cardiac murmur, the acoustic matching encapsulation design makes the auscultation more effective as well. Finally, as demonstrated by the experimental outcomes, the hollow concave ciliated MEMS sensor for heart sounds exhibited a 26.471 dB SNR, a value 5.238 dB higher compared to the electronic stethoscope from 3M Littmann.

## Figures and Tables

**Table 1:** Parameters for sensitive structures.

Structure Name	Parameter (mm)
Length of beams (L)	1 mm
Thickness of beams (t)	0.03 mm
Width of beams (W)	0.12 mm
Half-length of the center block’s side (a)	0.3 mm

**Table 2:** Material properties of photosensitive resin.

Material	Density (Kg/m^3^)	Poisson Ratio	Elastic Modulus (Pa)
Photosensitive resin	1.2 × 10^3^	0.41	4.2 × 10^9^

**Table 3:** Acoustic velocity values and characteristic acoustic impedances in varying media.

Medium	Density [ρ (Kg/m^3^)]	Acoustic Characteristic Impedance [z (Pa·s/m)]
Atmosphere (20 °C)	1.21	415
Water (20 °C)	998	1.48 × 10^6^
Blood	1055	1.656 × 10^6^
Soft tissue	1016	1.524 × 10^6^
Muscle	1074	1.684 × 10^6^
Medical coupling agent	950~1016	1.5~1.7 × 10^6^

**Table 4:** Heart-sound signal test data.

Parameter	First Heart Sound Duration (ms)	Second Heart Sound Duration (ms)	V_P-P_ (mV)	Background Noise (mV)	SNR (dB)
3M electronic stethoscope	130.125	95.5	1366.475	118.56	21.233
MEMS cardiac sound sensor	134.25	93.125	138.24	6.5625	26.471

## Data Availability

Not applicable.
